# Unmasking the Mask: Investigating the Role of Physical Properties in the Efficacy of Fabric Masks to Prevent the Spread of the COVID-19 Virus

**DOI:** 10.3390/ma14247756

**Published:** 2021-12-15

**Authors:** Adine Gericke, Mohanapriya Venkataraman, Jiri Militky, Hester Steyn, Jana Vermaas

**Affiliations:** 1 Department of Chemistry and Polymer Science, University of Stellenbosch, Stellenbosch 7600, South Africa; 2 Department of Material Engineering, Faculty of Textile Engineering, Technical University of Liberec, 461 17 Liberec, Czech Republic; jiri.militky@tul.cz; 3 Department of Sustainable Food Systems and Development, Faculty of Natural and Agricultural Sciences, University of the Free State, Bloemfontein 9300, South Africa; hesterjhsteyn@gmail.com (H.S.); NelJF@ufs.ac.za (J.V.)

**Keywords:** fabric masks, source control, filtration efficiency, air permeability, breathability

## Abstract

To function as source control, a fabric mask must be able to filter micro-droplets (≥5 µm) in expiratory secretions and still allow the wearer to breathe normally. This study investigated the effects of fabric structural properties on the filtration efficiency (FE) and air permeability (AP) of a range of textile fabrics, using a new method to measure the filtration of particles in the described conditions. The FE improved significantly when the number of layers increased. The FE of the woven fabrics was generally higher, but double-layer weft knitted fabrics, especially when combined with a third (filter) layer, provided a comparable FE without compromising on breathability. This also confirmed the potential of nonwoven fabrics as filter layers in masks. None of the physical fabric properties studied affected FE significantly more than the others. The variance in results achieved within the sample groups show that the overall performance properties of each textile fabric are a product of its combined physical or structural properties, and assumptions that fabrics which appear to be similar will exhibit the same performance properties cannot be made. The combination of layers of fabric in the design of a mask further contributes to the product performance.

## 1. Introduction

Face mask manufacturers are confronted with a lack of reliable information on the factors that determine the performance of fabric masks (generally referred to as “cloth masks”). This is especially disconcerting considering that face masks made of layers of textile fabrics have become part of the daily lives of many people all over the world. The design of a mask comprises a combination of the actual structure of the mask on the one hand, and the selection of layers of fabrics on the other. This study investigates the effect of fabric structural properties on the filtration efficiency (FE) and air permeability (AP) of masks, both considered crucial properties affecting the functionality of this type of mask.

Wearing of face masks made of ordinary textile fabrics (in addition to vigilant hand hygiene and social distancing) has been put forth as the most effective means to mitigate disease transmission by preventing outward transmission of contaminated droplets by an infected person [1,2,3]. This intervention was preempted by recommendations made by the World Health Organization (WHO), based on evidence that the COVID-19 virus is primarily transmitted between people through contaminated respiratory droplets (≥5 µm in diameter) and secondary contact routes [4]. The fabric masks are designed to act as “source control”, meant to provide sufficient filtration efficiency to stop the transmission of micro-droplets from an infected person to the environment [5].

Results from epidemiological and clinical studies assessing the effectiveness of mask-wearing indicate the importance of understanding how the components and design of a mask or face covering work together to reduce outward transmission of aerosols and droplets from respiratory excretions. Reports on the clinical effectiveness of masks or the role of fabric properties in the performance of masks are limited and findings are often conflicting [3,6,7,8]. For the future design of functional and effective fabric face masks, it is important to improve our current understanding of the principle of source control, the mechanisms of filtration through textile fabrics under the conditions pertaining to fabric masks, as well as the variations in fabric structural properties and their effects on performance properties.

Davies et al. [6] studied the ability of masks to reduce aerosol transmission (particles ≥5 µm) and found that the surgical mask had the highest filtration efficiency, and a mask made of a “tea-towel” had the second highest. Their study found that doubling the number of fabric layers improved the filtration efficiency (FE) but caused a significant increase in the pressure drop (indicating a low air permeability) measured across all three materials. Both the surgical mask and the homemade masks tested in the study reduced the total number of micro-organisms expelled during coughing. Considering the effect of the pressure drop on the comfort of the wearer, the “pillowcase” and “cotton t-shirt” fabrics were found most suitable for “improvised facemasks”. Anfinrud et al. [7] used laser light-scattering to detect droplet transmission during speaking and found that virtually no droplets were expelled while wearing a homemade fabric mask made from a “washcloth,” while significant levels were expelled without a mask. They concluded that “wearing any kind of cloth mouth cover in public by every person” (together with strict adherence to social distancing and hand hygiene) could significantly decrease the COVID-19 transmission rate. Konda et al. [8] evaluated a range of woven fabrics made from natural fibres. FE was calculated from particle upstream and downstream measurements and reported as a function of particle size (size ranges from 10 to 178 nm and from 300 nm to 6 µm). Comparing woven cotton fabrics with different thread counts, they found that woven cotton fabrics with increased thread count were preferable, with the “quilt” (2 layers of woven cotton with a nonwoven batting in between) providing excellent filtration (>90% for particles >300 nm). Hybrid combinations (e.g., 600 TPI cotton and 2 layers of chiffon) also performed well.

Although these findings are helpful and give an indication of the efficiency of fabric masks, the technical descriptions of the fabrics studied are limited and focus mainly on particles smaller than five microns, leading to questions about whether the findings can be successfully applied to fabric masks. Most of the publications are focused mainly on woven fabrics and natural fibres but lack information on knits, nonwovens, or synthetic fibres. The range of textile fabric options for masks is far more comprehensive than the examples studied, and manufacturers find it increasingly difficult to make informed choices when selecting fabrics for the design of masks.

The primary route of COVID-19 virus transmissions is most likely via small droplets ejected during speaking, coughing or sneezing, with the most common droplet size threshold between 5 and 10 µm [9,10,11]. Photographic imaging shows that masks can limit the spread of cough-generated particles [12]. Various studies on respiratory secretions report a reduced transmission of aerosol particles as well as “course” droplets (>5 µm) while wearing surgical masks [2,6,13,14]. Earlier studies assumed that coughing was mainly responsible for spreading droplets, but recent publications indicate that talking may be another key vector, with louder speech creating increased quantities and larger droplets. These factors are also associated with a higher viral load [7,9,14]. All the mentioned respiratory excretions are associated with a velocity much higher than that associated with normal breathing [6,11,15,16]. These findings have a significant impact on the performance properties of masks and should be kept in mind in the evaluation of the efficacy of masks.

While these studies have shown that masks can be effective, other studies suggest that masks made of certain fabrics can act as a source of infection themselves. Asadi et al. [3] studied masks and their components as potential sources of aerosol particles. It is well-established that fibrous cellulosic materials such as cotton or paper can release large quantities of micron-scale particles (mainly fibre fragments), referred to as “shedding”. Their study found that non-respiratory particles aerosolized from virus-contaminated materials can carry viruses and spread infection. It is postulated that particle emission from (homemade) fabric masks can substantially exceed emission compared to when no mask is worn, raising the possibility that shed fibre particulates from contaminated cotton masks might serve as a source of aerosolized fomites [3,17].

To understand the functionality and design of fabric masks it is important to differentiate between fabric masks worn by the public (to prevent the transmission of the COVID-19 virus) and speciality masks such as surgical face masks worn by medical professionals. High-efficiency surgical masks or respirators (also referred to as medical masks) are designed as personal protective equipment (PPE). They are required to filter small, aerosolized particles from the air during breathing to protect wearers against pathogens or infectious droplets in the air [4,18].

Fabric masks made of textile fabrics do not fall into the same category as surgical masks and are thus not considered suitable as (PPE). For a mask to fulfil its function, the fabrics it is made of must comply with two basic requirements. Firstly, the mask should provide an effective barrier against the transmission of droplets (≥5 µm) excreted through respiratory systems and resultantly traveling at a higher velocity than normal breathing, referred to as filtration efficiency (FE). Mask efficacy also depends on user compliance. This leads to the second requirement: the components of the mask should not inhibit the process of normal breathing and should maintain a state of thermo-physiological comfort in the facial area [19].

Understanding the mechanism of filtration is crucial to understanding the relation between the FE of a fabric and its structure, or that of a mask and its components. Filters for capturing aerosol particles are classified as “fibrous materials” or “porous membranes”, of which only the first applies to fabric face masks. In fibrous filters, fibres should be arranged perpendicular to the airflow. During the filtration process, particles collide or attach to the fibre surfaces. Particle penetration normally decreases exponentially with increasing filter thickness [20,21].

The theory of filtration considers five mechanisms by which an aerosol particle can be deposited onto a fibre in a filter, namely interception, inertial impaction, diffusion, gravitational settling and electrostatic attraction [8,22]. The first four refer to mechanical collection mechanisms in a fibrous mass, with interception and inertial impaction indicated as specifically applicable to the behaviour of larger particles in a fibrous structure such as a textile fabric. Interception occurs when a particle in a gas streamline comes very close to a fibre (less than one particle radius) and is captured on the fibre surface because of its finite size. Inertial impaction applies when a particle, due to its inertia, is unable to adjust quickly enough to the abruptly changing streamlines near the fibre, causing it to hit the fibre. Particles with bigger sizes are more easily captured as their high inertia prevents them from moving (with the air stream) around the fibres in a fabric or filter. Instead of flowing (with the air stream) through the fabric structure, particles with larger sizes collide with the fibres and can adhere to them. An increase in the velocity of the air stream will increase the kinetic energy as well as the inertia of the particles, which means that it can be expected that an increased airflow will increase the FE of a filter, especially for larger particles [20,23]. As stated, reported airspeeds for respiratory secretions from coughing and loud talking are much higher than that during breathing [11,15,24], and the particles or droplets involved are much larger than aerosols [11].

Fabric structure detail will determine the fibre arrangement, which relates to the filtration performance of the product as explained in the literature [20]. These physical fabric structural properties include fibre size, fibre surface structure, yarn properties (count, twist, and hairiness, etc.), yarn arrangement within the structure, fabric thickness (mm), count and weight (g/m^2^) and porosity.

The textile fabrics most often used in fabric masks can be described as woven, knitted, or nonwoven structures. In woven fabrics warp and weft yarns are interlaced at right angles according to a weave pattern. In both warp and weft knitted fabrics, yarns are inter-looped, forming a more flexible structure with a less isotropic fibre arrangement [25]. Knitted fabrics are not often used in filters, mostly due to the problem of stretching and potential distortion under stress, which can lead to a more open yarn arrangement and a resultant decrease in FE. It is recommended that this potential problem needs to be considered during mask design, where fitting issues or breathing can cause distortion [26]. Research has shown, however, that an FE performance similar to that of needle punch nonwovens can be achieved in engineered weft knit structures where stretching was minimized [27].

Nonwovens are fabric-like materials in which staple or filament fibres are arranged in a planar structure and bonded together by mechanical, heat or chemical treatment. The production of nonwovens proceeds in two steps, namely web (batt) preparation and bonding. Fabrics popular for masks are usually made of carded, air-laid, spunbonded (spun-laid) or melt-blown webs. A range of web bonding methods is possible. The thermal bonding process fuses individual fibres inside the web together or melts small areas of fibres on the surface together (point bonding). The sum of the bonded areas is usually limited to less than 5% of the total fabric surface area, leaving the rest of the fabric flexible and unaffected. The surface area percentage that remains unbonded will have a direct influence on the air permeability (AP) of the fabric, whereas the fibre arrangement will affect the FE. Spun bonded fabrics are usually bonded this way, but the method is also applied to other fibre web structures used in fabric masks [28]. Chemical bonding involves treating the batt with a bonding agent that strengthens the structure by bonding fibres together. This can also include the incorporation of synthetic latices, which can substantially affect the ability of a fabric to allow particles to be filtered [28]. In needle felting and hydroentanglement the fibres or fibre ends are bonded through frictional forces or fibre entanglement. The FE and AP are determined by the mas per area, thickness, fabric density, and fibre diameter and blend ratio [29]. Most of the fibre processing methods can be combined with any of the bonding methods, making the possibilities in manufacturing lines enormous and allowing a very large range of possible fabric performance properties. It is often found that two fabrics that appear similar in weight, thickness and porosity, can vary in FE [28].

After FE, the second requirement for masks to be effective is that the mask should not compromise the wearer’s normal breathing ability, even when worn for long periods. Fabric structural properties create a resistance to the air flowing through it (measured as a “pressure drop”). This “pressure drop” is directly proportional to the thickness of the fabric being measured. [20] In masks this relates to the ability of a wearer to breathe through the layers in the mask, with a high pressure drop indicating reduced breathability [4,8,20]. Fabric choices in the design of a mask, and particularly the selection of the filter layer, are crucial to ensure breathability. The same properties that determine FE also determine the air permeability of a fabric (AP), albeit in the reverse. This leaves the challenge to the mask designer to create the optimum balance between FE and AP.

As stated, the FE of a fabric depends on the fabric structural properties mentioned, as well as the size, velocity, and physical properties of the particles that a fabric is exposed to. Although many studies have been conducted on the efficacy of different fabrics for face masks, the effect of these parameters is still insufficiently explored. This can lead to mistakenly treating small differences between fabric structures as arbitrary. This argument is supported by Militky’s review on the topic [26]. A serious limitation in the reported literature is that generalizations (such as referring to fabrics in collective terms instead of using proper technical descriptions) can be misleading and can lead to misinterpretation or wrongful applications. This highlights the need for answers towards proper fabric selection and a more profound understanding of the topic.

Therefore, this paper aims to improve insight into the role that informed fabric selection can play in designing effective fabric masks. Scientific evidence will equip mask manufacturers and regulatory bodies with the necessary knowledge which can be applied to select and combine available fabrics to produce masks that perform optimally and comply with requirements.

## 2. Methodology

### Methods

The aim of this study was to investigate how fabric structures and layers of fabrics can inhibit the transmission of small particles in conditions that simulate those associated with respiratory excretions such as coughing, sneezing, or talking. No other method was available that measures filtration efficiency in conditions that simulates those associated with fabric face masks used as source control. A new method was developed to measure the filtration efficiency of ordinary textile fabrics, in single or multiple layers. The purpose is to measure the ability of the tested sample(s) to prevent the transmission of particles of 5 µm and larger. The method differs from the existing standard methods in that the velocity of the airstream was chosen to represent the velocity of air associated with the mentioned respiratory secretions, which is higher than the exhalations from normal breathing [11,15,24]. The method also measures the drop in airflow through the sample to indicate the expected “breathability” of a mask. It should be noted that the test method is not meant to replace the methods described in standards such as BS EN 14683:2019 (Medical face masks. Requirements and test methods.) that are currently used to certify medical masks. The principle of the test relies furthermore on the assumption that at an elevated air velocity and the filtration of random particles in the ambient air will behave comparative to water droplets of the same size as its behaviour will be based on physical characteristics rather than biological properties [6]. The equipment used was custom-built at in the textile laboratory at the University of Stellenbosch, in cooperation with industry, and further work is planned to optimise its efficiency and improve results. During the development of the test method, a selection of fabrics and fabric combinations (woven, knitted and nonwoven) was tested, and the results analyzed statistically using TIBCO Statistica software (TIBCO, Palo Alto, CA, USA) to confirm the reliability and repeatability of the procedure and equipment. The Welch ANOVA test confirmed that the means per repeat per sample did not differ at a 5% significance level and thus the test results can be considered repeatable.

It should be considered that the test method under discussion evaluates the FE of textile fabrics of varying yarn densities and fabric structures differing in stability and potential to be distorted under tension. Due to the end-use requirements, the test is carried out using a fairly high velocity airflow through the sample – creating pressure that might enhance fabric distortion. The standard deviations, and, resultantly, the coefficient of variation for each, will be larger than can be expected from more stable substrates – especially when single layers of fabric are tested. The fact that fabric face masks do not fit as tightly as respiratory masks and will allow air to escape through the open sides, emphasizes the fact that the FE values provided in this method, should be used a tool to differentiate between fabrics with good and poor FE, and not necessarily measure small differences between samples.

**Figure 1:**
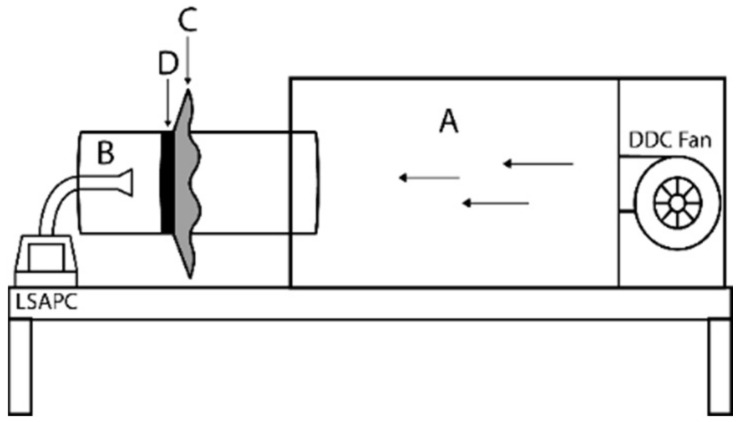
Experimental setup to measure FE and AP of test specimens.

The experimental apparatus (Figure 1) consisted of an air sample generation chamber (A) and air collection chamber (B), with the test specimen (C) mounted inside a tube connecting the two chambers. Chamber C is used as a wind channel through which a controlled stream of ambient air is directed towards and through a mounted fabric sample, secured with a clamp (D) to prevent leakage. The principle of the test is to determine the number of particles transmitted through the sample to calculate its barrier efficiency. A light scattering airborne particle counter (LSAPC) is used to count the size and concentration of airborne particles at designated sampling locations. The typical size range of particles measured is between 0.1 µm and 10 µm in particle size, although the focus was on those that measure 5 µm. The air velocity (in the wind tunnel as well as downstream) is measured with a hot-wire anemometer (Testo 425, Testo North America, West Chester, PA, USA) and used to calculate the airflow resistance of the fabric sample tested.

During the test procedure, a controlled flow of ambient air is directed through a wind channel (A) at 3.5 m/s. Before testing the sample fabric, the particle content of the ambient air (pc_amb_) is determined by using the experimental setup without a fabric sample. Thereafter the sample fabric is mounted as shown in Figure 1 and the particle content of the ambient air is collected downstream of the sample (from chamber B) and analysed with the LSAPC to record the number of airborne particles not filtered by the sample (pc_sample_). The number of particles in the air (pc_amb_) is used as a reference to calculate the filtration efficiency (FE) of the sample.

The difference in the air velocity in the wind tunnel is measured on both sides of the mounted sample to indicate the air permeability (AP) of the fabric sample.

(1) $$\mathrm{Calculations}:\;[BE\;\left(\%\right)=\frac{{\mathrm{pc}}_{\mathrm{amb}}-{\mathrm{pc}}_{\mathrm{sample}}}{{\mathrm{pc}}_{\mathrm{amb}}}\times 100]$$

(2) $$\mathrm{and}\;\left[AP\;\left(\%\right)=\frac{air\;velocity\;\left(after\;sample\right)}{air\;velocity\;\left(before\;sample\right)}\times 100\right]$$

[where pc_amb_ = number of particles (5 μm) measured in ambient air and pc_sample_ = number of particles (5 μm) not filtered by the sample. Air velocity is indicated in m/s].

All fabrics were characterized according to their physical structural properties. Scanning Electron Microscope (SEM) and light microscopic imaging were obtained to closely examine the physical structural characteristics of the tested fabrics that could affect or explain results. Fabric density is indicated as fabric count for woven fabrics (total yarns per cm^2^), wales per cm (wpc) for weft-knits and courses per cm (cpc) for warp-knits. The term yarn type refers to whether the yarn consisted of staple (spun) or filament fibres. Fabric thickness (mm) was measured with a John Bull Imperial Indicator. Fabric Weight (g/m^2^) was determined according to SANS 79, using a circular cutter to ensure accuracy. Results were used to calculate fabric porosity as follows:

(3) $$\left[P=1-\frac{\rho_{\mathrm{fabric}}}{\rho_{\mathrm{fibre}}}\right]$$

[where P from fabric density ρ_fabric_ (kg/m^3^) and fibre mass density ρ_fibre_ (kg/m^3^) [30]].

## 3. Materials

The FE and AP of a wide range of commonly available textile fabrics, regarded as potentially suitable to be used in face masks, were measured as described. Given the lack of reliable, scientific information on the topic, this was originally meant as an exploratory study to broaden the bank of knowledge on the potential performance of the textile fabrics and combinations thereof for use in the described fabric masks. The focus was mainly on FE and AP. Fibre compositions included polyester (P), cotton (C), polyester/cotton (PC), polyester/viscose (PV), polyester/acrylic (PA) and polypropylene (PP). All fibres, except for polyester microfibers (P_mf_), were of regular size. The textile fabrics selected were characterized by structure (woven, knitted or nonwoven), density (fabric count), aerial weight (g/m^2^), thickness (mm), fibre type, yarn type and porosity (%). Fabric counts were grouped into 4 categories as described in Table 1 to facilitate statistical analyses.

## 4. Results and Discussion

**Figure 2:**
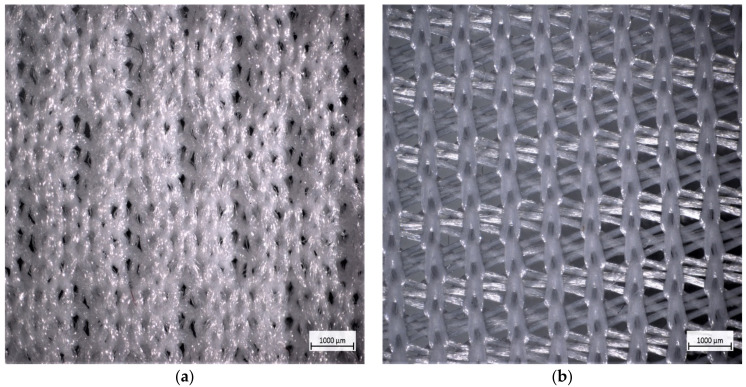

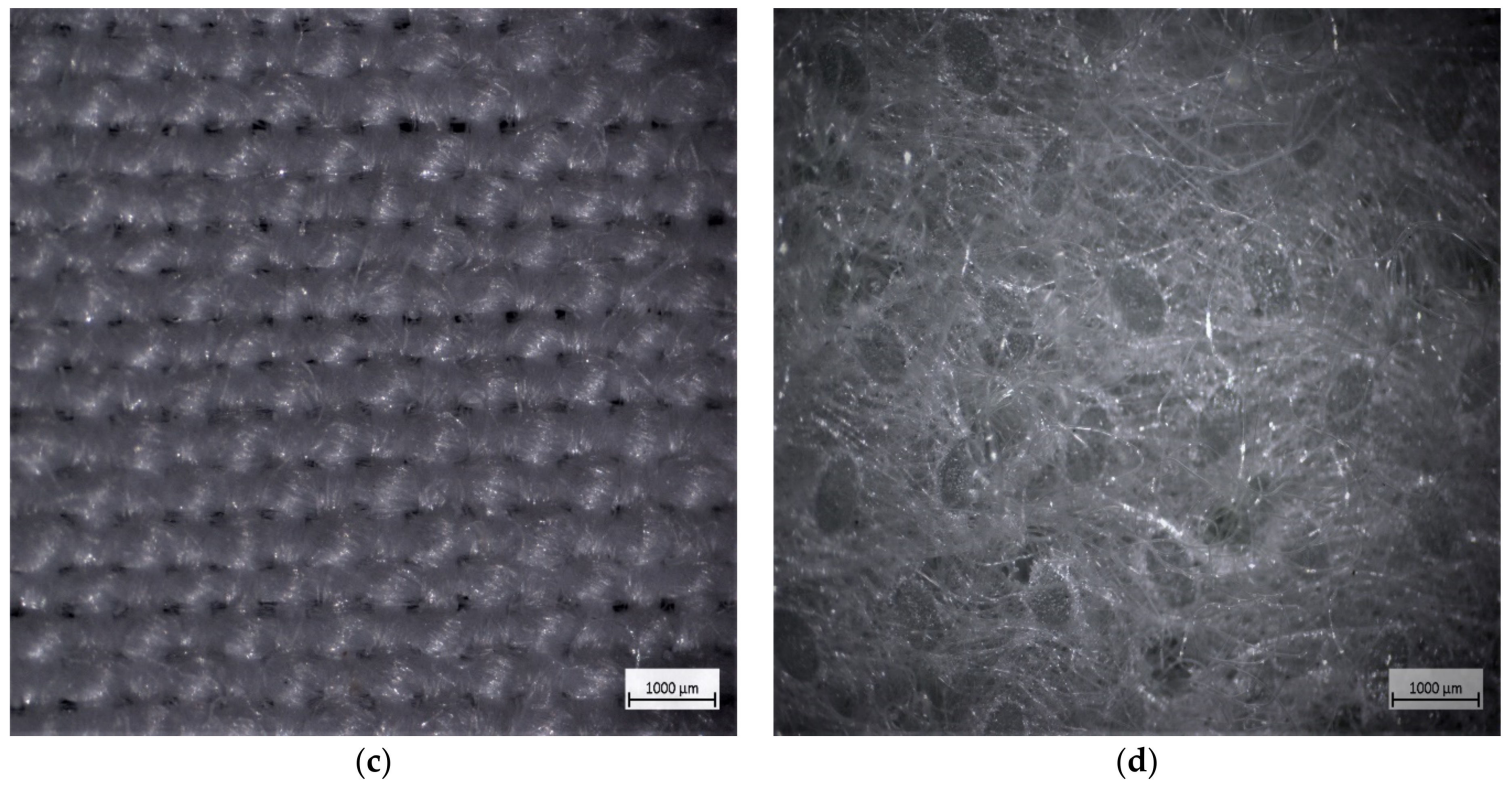
Light microscopic images of weft knit, warp knit, woven and nonwoven fabrics (scale bar 1000 μm). (**a**) Weft knit eyelet design, (**b**) Warp knit, (**c**) Woven (plain weave), (**d**) Nonwoven fibre web.

**Figure 3:**
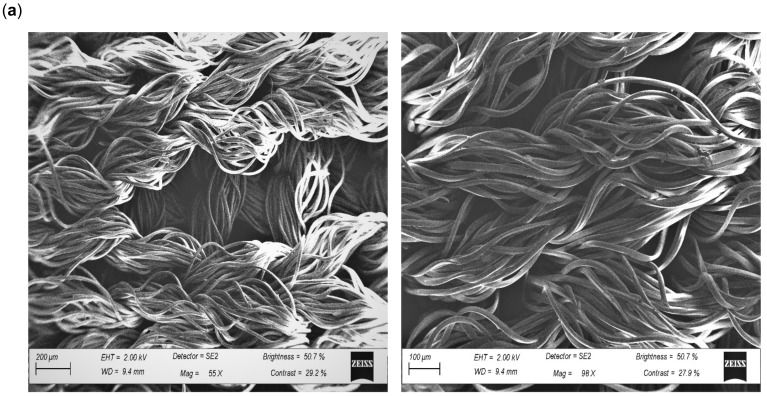

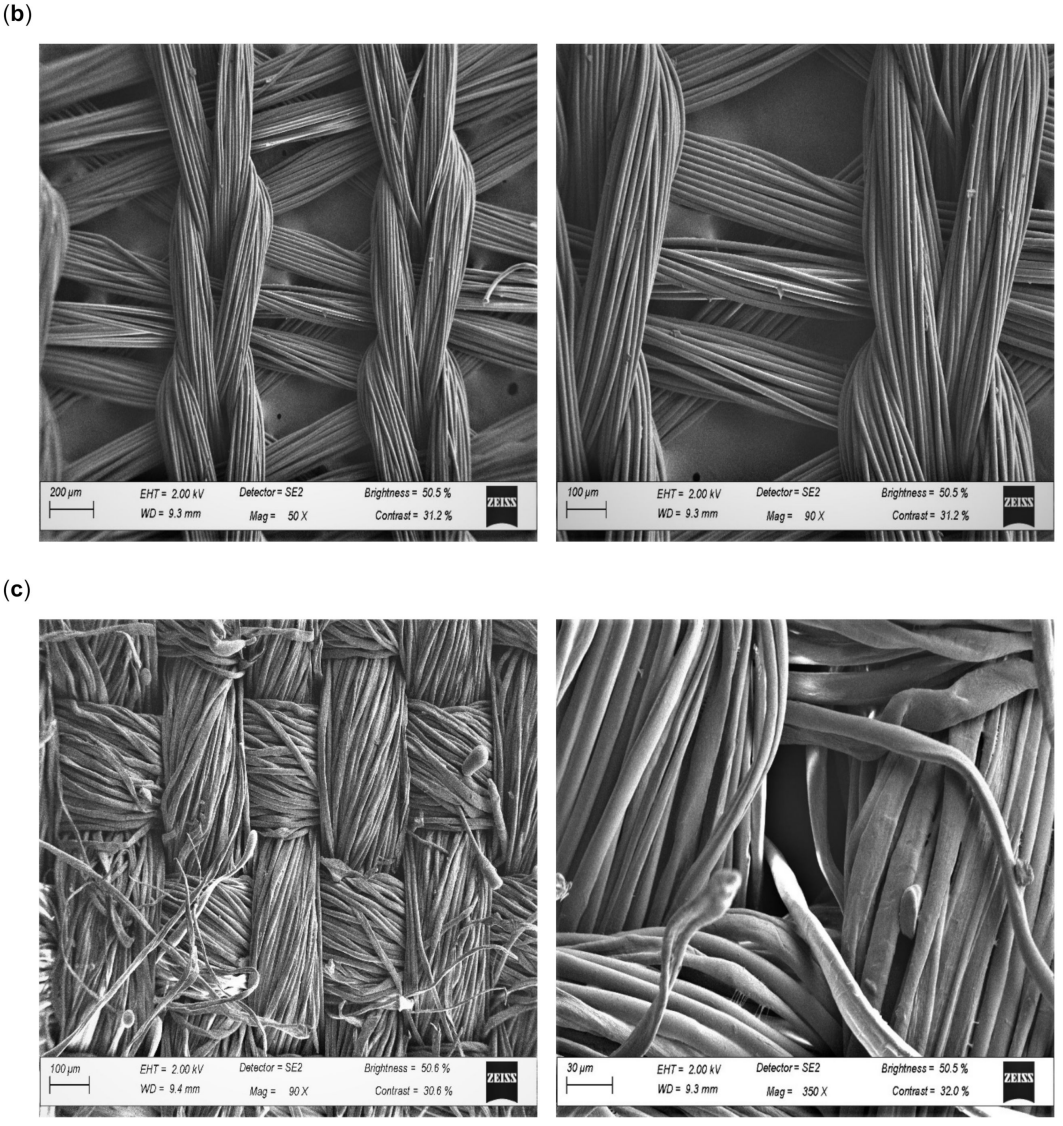

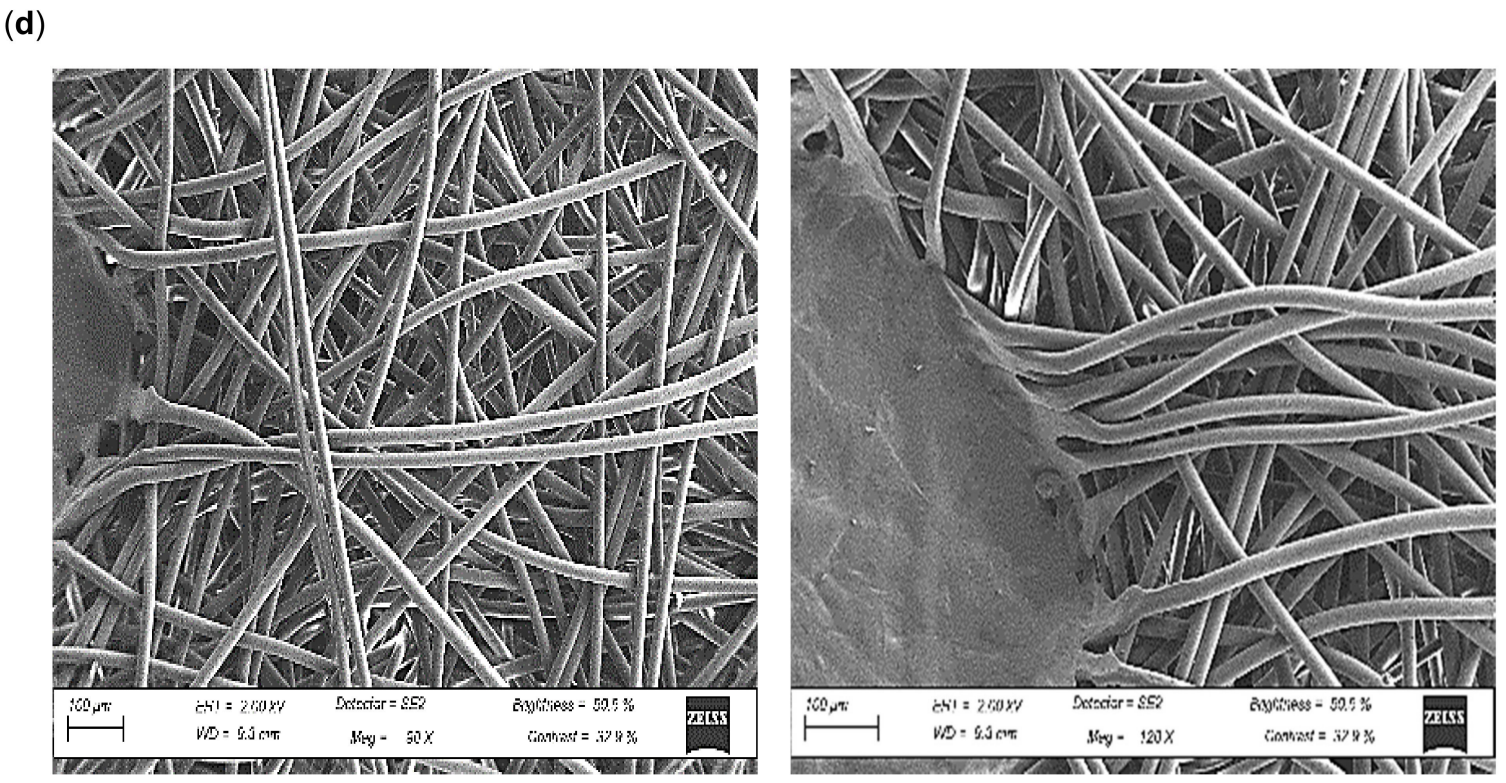
SEM images of weft knit, warp knit, woven and nonwoven fabrics. (**a**) Weft knit (eyelet design), (**b**) warp knit (tricot), (**c**) Woven (plain weave), (**d**) Nonwoven point sealed spun-bond.

**Figure 4:**
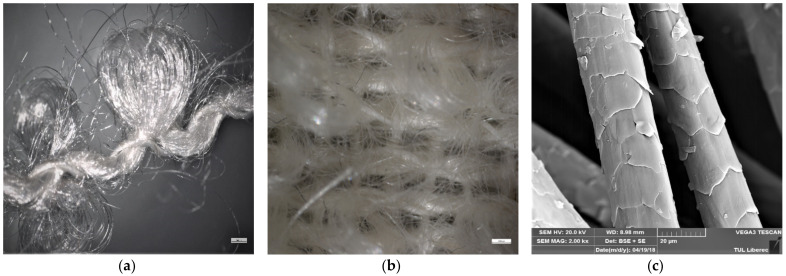
SEM images of (**a**) mohair boucle yarn (scale bar 200 μm), (**b**) mohair boucle weave fabric (scale bar 1000 μm) and (**c**) mohair fibre surfaces (scale bar 20 μm).

The data from the range of selected fabric samples were analysed and the FE (%) and AP (%) were calculated as described in the methods. Results were divided into three groups, namely woven, knitted and nonwoven (filter) fabrics. Results on single layers, double layers and three layers (double layers combined with a filter layer) of woven and knitted fabrics, as well as single layer nonwovens, are presented in Table 2 and Table 3. TIBCO Statistical Software was used to investigate the effect of the number of layers and fabric structural parameters on FE and AP. The SEM and microscopic imaging of fabric and fibre structures are shown in Figure 2, Figure 3 and Figure 4.

The microscope images give a clear indication of the yarn or fibre arrangement on the fabric surfaces, but a better explanation can be found in the SEM imaging of the fibres at different magnifications.

Initial observation of the weft knit fabric surface images in Figure 2 and Figure 3 creates the perception of a structure that is much more “open” than that of the woven fabrics. Further inspection reveals a multitude of layers of fibres randomly arranged at different levels, putting them in the perfect position to act as a filter. The unexpectedly high FE of the more porous knitted fabrics (refer Table 2) can be explained based on the theory on inertial impaction, during which a particle in an airstream is captured on the surface of a fibre (positioned perpendicular to the direction of the airflow) due to its inertia or inability to move around the fibre. This is more pronounced when more than one layer was tested, increasing the number of fibres arranged perpendicularly in the path of the airstream, without compromising the porosity and thus AP.

For a better understanding of the effect of fibre arrangement and fabric structure on FE and AP results, light microscope and SEM images of fabric structures were studied and can be seen in Figure 2, Figure 3 and Figure 4.

The same reasoning can be applied to the observed fibre arrangement in the nonwoven fabrics in Figure 2d and Figure 3d, explaining why both the FE and AP of the nonwoven fabrics (as shown in Table 3) are in the same range as that of the knitted fabrics. The point sealed areas provide a total block to particles and air, which means that size of the unsealed surface will determine FE and AP.

It can be observed from the high magnification images of the woven fabric in Figure 3c that although the packing of the yarns appears to be tight, the interstitial spaces at an interlacing can be quite large and would easily allow microdroplets of 5 µm and even larger to move through. The problem can be overcome by adding more fabric layers. However, it should be considered that interstitial openings between fibres or yarns are the only route to allow air to permeate through the fabric structure, explaining the low AP results reported on woven fabrics and the effect of weave density on AP (Table 2). The low AP will have a direct influence on the ability of a wearer to breathe comfortably through a mask, especially when more than one layer is involved. The smooth filament yarns and open structure of the 100 g/m^2^ warp knit fabrics in Figure 2b and Figure 3b explain why this fabric provides little resistance to airflow as well as to particle penetration.

Due to the unique characteristics of boucle yarns, woven boucle weave fabrics exhibit a highly random fibre arrangement as can be seen in the SEM images in Figure 4a,b. Mohair fibres also have a distinctive scaled surface (Figure 4c) that differentiate them from most other fibres. Both these characteristics could attribute positively towards the ability of the fabric to filter small particles through the mechanism of interception or inertial impaction without compromising breathability. This initiated a focus on the FE and AP of the boucle weave mohair sample that was included in the sample set. It was found that the FE (76%) and AP (94%) of the single layers tested were comparable to that of most of the nonwoven fabrics that were considered acceptable for filters. With the increase in the usage of masks, a removable filter with a composition of 100% natural fibres would be an interesting option to be considered instead of the available synthetic alternatives.

**Figure 5:**
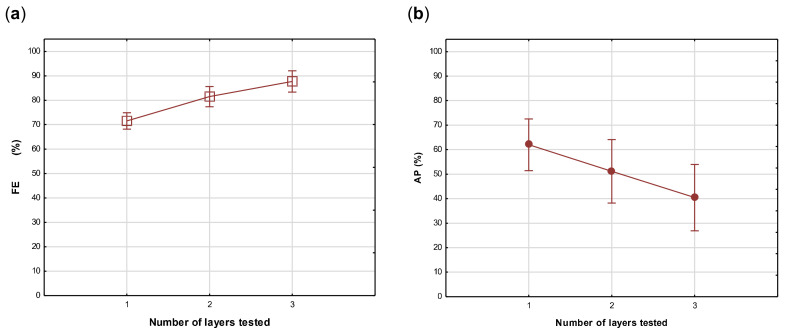
Analyses of variance showing the effect of the number of layers tested on filtration efficiency (FE) (**a**) and air permeability (AP) (**b**).

In the statistical analyses of the data, a one-way analysis of variance (ANOVA) was calculated on the effect of the number of layers on the FE and AP of all the fabrics tested. The analyses depicted in Figure 5a show a significant increase in FE from one to three layers (Vertical bars denote 95% confidence levels). The Bonferroni test confirmed highly significant effects (*p* < 0.01) between one and two, as well as two and three layers for FE. It was expected that the AP would decrease similarly with an increase in the number of layers, but it is clear in Figure 5b that although a steady decrease was noted from one to three layers, it was not statistically significant (*p* > 0.1), creating interest for further investigation into the effect of fabric structural parameters on AP and how this can be manipulated in the selection of fabrics to improve the breathability of the masks without compromising FE.

**Figure 6:**
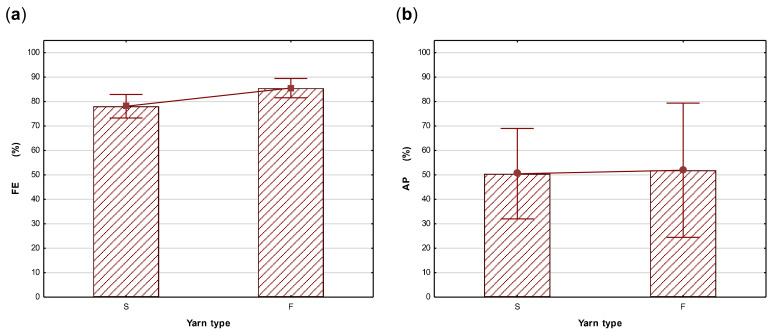
A comparison of the mean FE (**a**) and AP (**b**) of fabric samples according to their yarn type (filament or spun from staple fibres). (Vertical bars denote a 95% confidence level).

The Spearman’s rank-order correlation test was used to measure the strength and direction of associations between fabric structural properties and fabric performance properties (FE and AP). Although it was expected that factors such as a higher fabric weight and thickness and a lower porosity would improve FE, no significant correlations were indicated in the tests on single layers of woven, knitted, and nonwoven fabrics, confirming these assumptions cannot be made. In the tests on two layers, a significant correlation was indicated between yarn type and FE (R = 0.49, *p* < 0.05), with fabrics made of multifilament yarns having a higher FE than those from spun (staple) yarns. This contradicts the assumption that the yarns spun from staple fibres usually have a “hairy” surface and would produce a product with an improved FE due to the effect of the protruding ends. The results could be due to the close packing of the finer filaments which prevents droplets from moving through interstitial spaces in yarns. Although not statistically significant, this was also noticed as a trend in the single layers, where the correlation was less pronounced than when two layers of fabric were tested. The AP and FE mean values for spun versus filament yarns are compared in Figure 6a,b. Yarn type (staple or filament) did not affect AP.

The Spearman R and *p*-values calculated on the AP values indicate significant correlations between fabric structure, fabric thickness (and resultantly porosity) as well as fabric count (in the woven fabrics) and AP in the single, double, and triple layer knitted and woven fabrics (*p* < 0.05). In the nonwoven fabrics, no correlations were indicated between fabric structural properties and AP.

**Figure 7:**
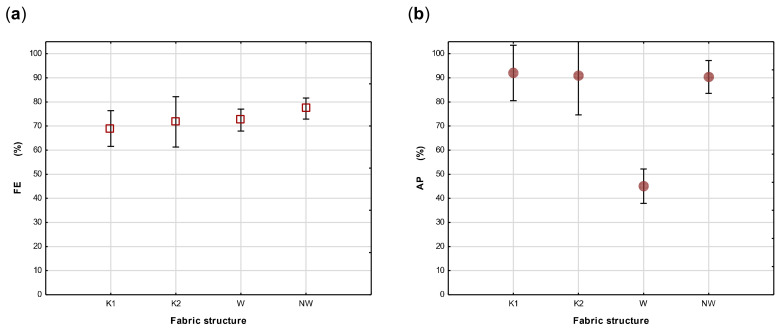
Anova calculated on the effect of fabric structure on FE (**a**) and AP (**b**) of knitted (K1 and K2), woven (W) and nonwoven (NW) fabrics.

Although the researchers acknowledge that the use of single layers in cloth masks are not recommended [19,31], the test results on single fabric layers were used to provide insight into the behaviour of the selected fabrics concerning FE and AP. The Anova, calculated on the effect of fabric structure on FE and depicted in Figure 7a, indicates that the effect of the basic structure of a fabric, (whether it was woven, knitted or nonwoven) on its FE was not significant (*p* > 0.05) and that none of these structures can be considered a better choice than the others concerning FE. The low AP of the woven fabrics in comparison with the knitted fabrics and nonwovens (*p* < 0.05) are depicted in Figure 7b.

**Figure 8:**
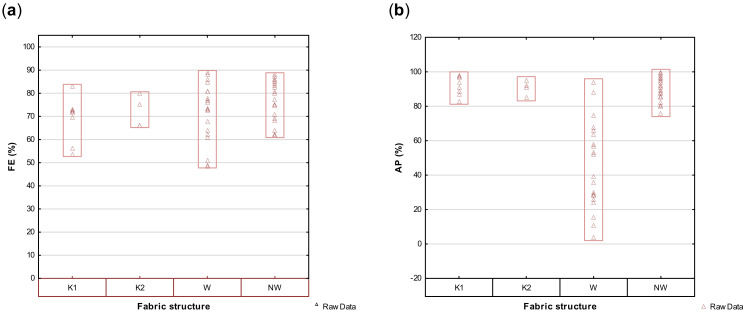
Variability plots comparing the variance in FE (**a**) and AP (**b**) of knitted (K1 and K2) woven (W) and nonwoven (NW) fabrics.

Variability plots on the effect of fabric structure on FE and AP were employed to highlight the performance of individual fabrics within the fabric structure groups and provide valuable information on the data spreads from which the means were calculated. A wide variation in the FE of weft knitted (K1) and woven (W) fabrics as well as a considerable variance in the AP of the single layer woven fabrics (in contrast with the knitted or nonwoven fabrics) is illustrated in Figure 8a,b. This confirms the earlier postulation that the FE performance of woven and knitted fabric structures, as well as nonwoven filters, cannot and should not be generalized as it could lead to false assumptions or conclusions. The lack of significant correlations between the structural properties and FE shows that in any fabric structure, the unique combination of all these structural properties will determine the performance of the product. Furthermore, the unique combination of layers of fabric in the design will also contribute to the final product performance.

**Figure 9:**
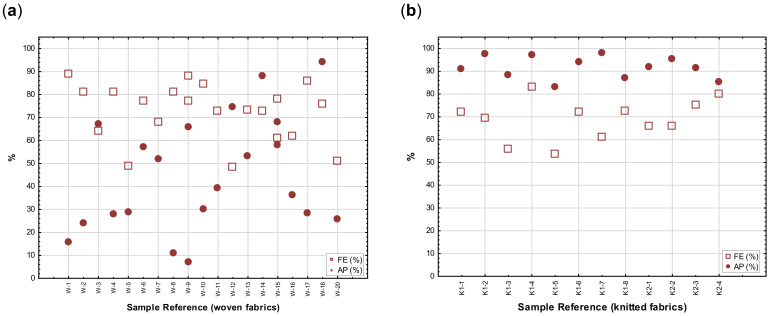
Scatterplots depicting the FE and AP of single layers of (**a**) woven and (**b**) knitted fabric samples.

**Figure 10:**
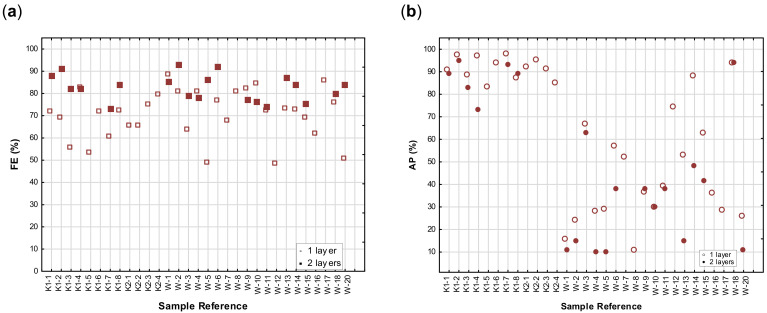
A comparison of least squares means for FE results of the single layer or double layer fabrics on FE (**a**) and AP (**b**) of individual woven and knitted fabrics.

As a result of the exploratory nature of the study, it is worth discussing the interesting observations within the individual fabric structural groups. The FE and AP of individual fabric samples within the fabric structure groups will subsequently be depicted in the scatterplots in Figure 9a (woven fabrics) and 9b (knitted fabrics) to illustrate the variations between fabrics. The difference in the FE and AP of single and double layers are shown in Figure 10a,b. Even though the fabrics were sorted according to basic fabric structure, fabric weight and fibre type, no specific trends can be seen, emphasising the unpredictability of especially the FE of the fabrics and its dependence on individual structural detail, which will be discussed below. The main observation is the noticeable difference in the overall performance of the knitted and woven fabrics.

For the single layer woven fabrics, the FE varied between 45% and almost 90%, with almost 50% of the fabric samples having an FE of 75% or more. The highest FE were measured on the polyester fabrics containing microfibres and those where the weave count was exceptionally high, indicating the effect of closely packed small fibres and yarns with very small interstitial spaces. This confirms findings by Konda et al. [8] It was noted that the plain weave 125 g/m^2^ polycotton, as well as a few others in the very high weight range, also exhibited a FE of higher than 75%.

The low AP measured on most of the woven, as opposed to the knitted fabrics, should be noted in Figure 9b, pertaining particularly to the higher count plain weaves as well as those with higher planar weights. Only three of the twenty-three results in the graph (Figure 10b) had an AP of more than 75%. The AP decreased even further when an extra layer was added.

To ensure an acceptable FE, it is recommended that fabric masks should be made of at least two or three layers of fabric [4,19]. The FE of single and double layers is graphically compared in Figure 10a, highlighting firstly that in all cases the addition of a second layer drastically improved and secondly how unpredictable the FE of single fabric layers are. This justifies recommended guidelines by the WHO and others [4,19].

Knitted fabrics are generally not recommended for masks, mostly because it is expected that the open structure and stretchability of knitted fabrics would not allow for an acceptable FE. The results on the weft-knitted fabrics proved this assumption wrong. Acceptable FE values, comparable to that of woven fabrics, were achieved in more than half of the single layer fabrics (Figure 9b). As in the case of woven fabrics, performing the tests on two layers improved the FE of the knitted fabric samples (Figure 10a). This was unexpected, as the FE of the majority of the knitted fabrics exceeded 78% and even surpassed that of the woven fabrics in many cases.

Furthermore, the AP of more than 80% was achieved in most of both the warp and weft knitted constructions, which would be highly advantageous in masks that have to be worn for long periods. When two layers were tested, no significant decrease was noticed and none of the measurements was below 85%. This can be attributed to the knitted structures having a more open structure and higher porosity. The data on the AP of knitted fabrics is graphically depicted in Figure 9b and Figure 10b. These results indicate that two layers of knitted fabrics can be used with success in a fabric mask to achieve an acceptable FE result, without compromising the breathability of the mask. Care was taken during the tests to prevent stretching of the knitted samples, as distortion under tension leads to a more open structure and decreased FE. This problem can be addressed in the construction of masks by adding a third layer (e.g., a nonwoven fabric with high AP) for stability.

**Figure 11:**
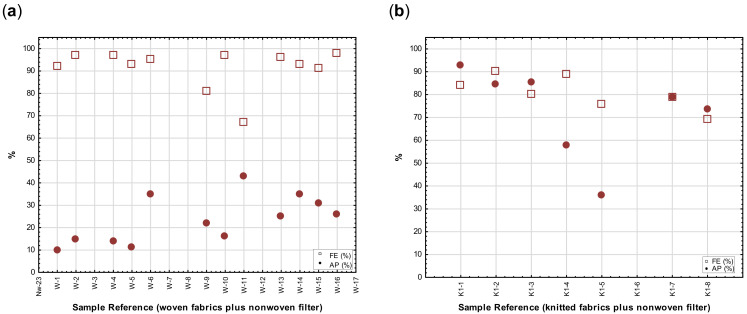
Scatterplots depicting the FE and AP of three fabric layers, consisting of two layers of woven (W-1 to W-17) fabrics (**a**) plus a nonwoven filter layer or two layers of weft knitted (K1–1 to K1–8) fabrics (**b**) plus a nonwoven filter layer.

To investigate the effect of adding a third or filter layer on the expected AP and FE of a mask, two fabric layers were combined with a third filter layer. The same filter fabric was used in all the tests, consisting of a spun-bond layer, combined with a fibre web in a needle punch process and finished with an acrylic binder (weight 100 g/m^2^). Results are depicted in Figure 11a,b.

It was found that in both the woven and knitted sample sets, a FE of more than 75% was measured when three layers were combined. The AP in the woven fabric combinations was much lower than in the knits, where the breathability was maintained at an acceptable level.

An observation worth noting is that during the execution of the tests, an unrealistically high particle count (pc_sample_ > pc_amb_) was often recorded when fabrics containing cotton were tested, rendering unrealistic results. This was accelerated by the elevated pressure that developed in the very tightly woven or knitted fabrics due to the high air velocity in the test chamber that caused shedding or breaking off of small pieces of fibre (or even accumulated dust) in the sample. This finding justifies the named concerns and confirms postulations that shedding is possible and that it can cause the spreading of infection through contaminated fibre fragments [17]. The problem was more often found when multiple layers provided increased resistance to the air stream directed through it. For this reason, the data summary (Table 2) does not include many cotton fabrics.

Recommended guidelines for the design of masks strongly suggest the inclusion of a third (filter) layer (either as part of the construction or removable insert) in the design of masks to improve the FE [4,19]. Nonwoven constructions are usually preferred because of the high air permeability and presumed FE [19]. To evaluate the efficiency of different filter materials to be used as recommended in fabric masks, a wide selection of nonwoven fabrics, differing in weight, thickness, as well as bonding method, were tested in a single layer to compare the FE and AP.

**Figure 12:**
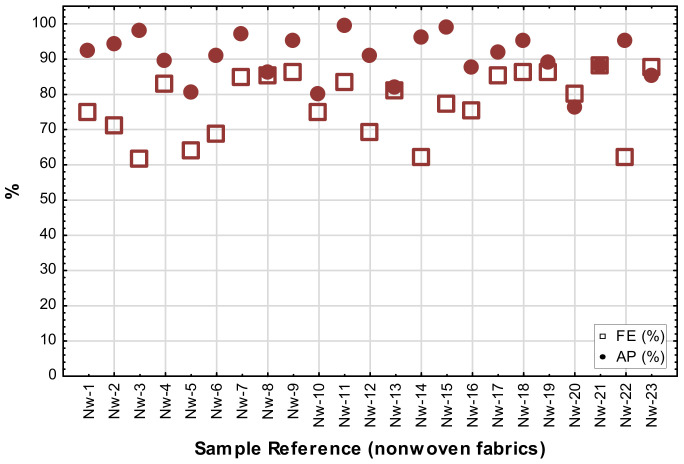
Scatterplots depicting the FE and AP of single layers of nonwoven fabrics (Nw-1 to Nw-23).

Although fabrics are grouped according to construction method (mainly bonding method) and weight, no specific trends were noticed, as can be seen in the FE or AP values shown in Figure 12. Contrary to expectations, no correlations exist between the nonwoven fabric structural properties (porosity, thickness, and weight) and the predicted AP, as confirmed by the Spearman rank-order tests. The scatterplot in Figure 12 illustrates the consistently high AP of the nonwoven fabrics. All the AP values were above 75% with more than half of them exceeding 90%. This makes these structures excellent for filtration purposes in filters, as the addition of a third layer or filter insert in the mask design can significantly improve the FE but does not have a detrimental effect on the breathability of the mask.

From the literature study, it was expected that the bonding method applied in the manufacturing of a nonwoven might affect its FE, but as mentioned above, no specific correlations were found between the bonding method and FE in this sample set. Most of the point sealed spun bond fabrics performed very well, but some exceptions should be noted. In the case of the other fibre webs with point seal as bonding method, the FE results were consistently above 75%. As expected, the sample with the highest weight performed the best, because this implies a higher concentration of fibres within the structure. This was specifically evident in the needle punch fibre webs, where fabric weight was an important parameter influencing FE. The needle punch fabrics with fabric weight ≥ 100 g/m^2^ were equal in FE to that of the spun bond point sealed ones of similar weight. The FE of the binder bond fabrics was more unpredictable, confirming the prediction in the literature that a variation in chemical binders applied during the fabric production process can lead to FE results that cannot be explained from observed structural information only. The FE of a fabric relies on its ability to obstruct and hold small particles moving through its structure [20], explaining the importance of fibre arrangement and the presence of chemical globules, synthetic lattices or bonded fibres in the performance properties of nonwoven structures.

Although nonwoven fabrics were at first mainly considered for filters, spun-bond point sealed fabrics have been used successfully as first (skin-contact) layers in fabric masks, as well as in double or triple layer designs. Three of the five spun bond point sealed fabrics performed excellently with a FE above 80%. The other two, which had comparable weight descriptions, but were from different manufacturers, did not perform as well. The unpredictability of these fabrics can be ascribed to finishing treatments such as washing or finishing (that the consumer may be unaware of), which can have a detrimental effect on FE due to the vulnerability of the structures to distortion or damage. The fact that spun bonds are often produced in very low weight ranges emphasizes that care should be taken during the production and finishing of masks, but also during wash cycles at home, not to damage the structure or compromise their ability to filter small particles or droplets.

## 5. Conclusions

The results of this study confirm a significant improvement in FE when the number of layers is increased to more than one. This shows that, in general, the FE of one layer of fabric should not be regarded as sufficient. This serves as justification for regulations and guidelines that were published at the onset of the pandemic in 2020. The woven fabrics (in both one and two layers) had, in general, the highest FE. However, when two layers of fabric were combined with a (filter) layer, an FE of more than 80% was achieved in almost all cases for both the knitted and woven fabrics.

Although an attempt was made to determine whether any specific physical fabric properties contributed more than the others to improve FE, little or no correlations were found. None of the fabric structures could be singled out as having a more pronounced influence than the others. It should be emphasized that the variance in results achieved within the sample groups confirms that the FE of woven and knitted fabric structures, as well as nonwoven filters, cannot and should not be generalized. In any fabric structure, the unique combination of all the named physical structural properties determines the performance properties of the product. Furthermore, the unique combination of layers of fabrics in the design of a mask also contributes to the final product performance.

Mainstream recommendations often advise against the use of knitted fabrics in masks. Surprisingly, it was possible to achieve a FE of up to 80% without a drastic loss of AP in certain two-layer assemblies of weft-knit fabrics. This finding raised a new awareness of the potential of knitted fabrics in the design of fabric masks, despite previous reservations. Double layer knitted fabrics, especially when combined with a third filter layer, can provide an FE comparable to that of woven fabrics, but without compromising comfort concerning the breathability of the mask. The high FE, despite the high AP found in specifically the eyelet design weft-knitted polyester, can probably be ascribed to the effect of inertial impaction based on the multilayer fibre arrangement in the knitted structure. Care should however be taken to prevent distortion due to stretching or elongation of the knitted fabrics under tension. This can be done successfully with the inclusion of a breathable nonwoven layer that provides dimensional stability.

To maintain a state of thermo-physiological comfort and enhance “user-compliance” it is important that the wearer of a mask must be able to breathe normally. This finding highlights a potential problem that should be addressed during the design of a mask. The low AP of many of the woven fabrics should be a serious concern in designing functional masks for long term wear. It should also be noted that in multi-layered masks, the inclusion of one layer with an unacceptably low AP can render the whole product dysfunctional.

Based on their FE and AP properties, nonwoven fabrics are often recommended as a third (filter) layer in fabric masks. Nonwoven fabrics are comparable to the knitted structures in both FE and AP. For this reason, nonwovens with acceptable sensorial and moisture management properties, such as point-sealed spun-bonds, can also be considered as inner layers and in some cases even outer layers in fabric masks. However, for multiple-use masks, it is important to note that the nonwovens can be susceptible to distortion due to abrasion during use or multiple cleaning or disinfection cycles which can cause a decrease in FE. The problem can be overcome with mask design that allows for nonwoven filters to be removed and disinfected with hot water instead of being sent through multiple wash cycles.

The results of this study have illuminated a few general trends, but importantly also exceptions in the effect of fabric structural properties on FE and AP. This emphasizes the importance of the fact that each textile fabric is a product of its combined physical properties. This makes every fabric unique, justifying the postulation that generalizations should not be made based on assumptions that fabrics that appear to be similar will exhibit the same performance properties.

## Figures and Tables

**Table 1:** Summary of structural variations in the sample set and references used in this paper.

Fabric Structure		Fibre Type		Yarn Type		Fabric Density ^#^	
Description	Ref.	Description	Ref.	Yarn Type	Ref.	Categories	Ref.
Plain weave Twill weave Weft knit. Warp knit. Spun bond (point sealed) Fibre web (binder bond) Fibre web (needled)	W pw W twill K1 K2 Sb(ps) Fw(bb) Fw(n)	Polyester P(microfibre) Cotton Polycotton Poly-acrylic Polypropylene Mohair	P P_mf_ C PC PA PP Mh	Spun (staple) Filament Textured-filament	S F FT	(yarns/cm^2^) 106–135 76–105 46–75 10–45	A B C D

^#^ fabrics were grouped in categories according to weight or count for ease of analysis.

**Table 2:** Fabric structural detail and results of knitted and woven fabrics tested in single, double and triple layers.

Sample Ref.	Sample Description	Layers Tested	Fabric Structure Detail							Results		Layers Tested	Results		Layers Tested	Results	
			FibreContent	Yarn Type	Thickness (mm)	Structure	Weight (g/m^2^)	Count (Categorized)	Porosity (%)	FE (%)	AP (%)		FE (%)	AP (%)		FE (%)	AP (%)
**WOVEN FABRICS**																	
W-1	W100 P_mf_ pw	1	P	F	0.20	W	100	B	64	89	16	2	85	11	3	92	10
W-2	W110 P pw	1	P	F	0.25	W	110	B	68	81	24	2	93	15	3		
W-3	W115 P pw	1	P	S	0.25	W	115	C	64	64	67	2	79	64	3	97	15
W-4	W115 P pw	1	P	F	0.25	W	115	B	67	81	28	2	78	10	3	97	14
W-5	W150 P_mf_ pw	1	P	F	0.25	W	140	C	75	49	29	2	86	10	3	93	11
W-6	W200 P_mf_ pw	1	P	F	0.30	W	200	C	43	77	57	2	92	38	3	95	35
W-7	W225 P pw	1	P	F	0.30	W	225	D	35	68	52	2	*		3	*	
W-8	W250 P pw	1	P	S	0.40	W	250	D	55	81	11	2	*		3	*	
W-9	W115 PC pw	1	PC	S	0.20	W	115	B	65	77	66	2	77	38	3	81	22
W-10	W125 PC pw	1	PC	S	0.20	W	125	B	65	85	30	2	76	26	3	97	16
W-11	W175 PV pw	1	PV	S	0.32	W	175	D	51	73	39	2	74	38	3	67	43
W-12	W225 PV pw	1	PV	S	0.40	W	225	D	61	49	75	2	*		3	*	
W-13	W125 C pw	1	C	S	0.20	W	125	A	68	73	53	2	87	15	3	96	25
W-14	W150 C pw	1	C	S	0.40	W	150	D	51	73	88	2	84	48	3	93	35
W-15	W175 C pw	1	C	S	0.40	W	175	C	72	61	58	2	68	34	3	91	31
W-16	W225 C twill	1	C	S	0.40	W	225	C	64	62	36	2	*		3	98	26
W-17	W225 C pw	1	C	S	0.40	W	250	C	60	86	29	2	*		3	*	
W-18	W250 boucle	1	Mh	S	5.20	W	250	D	97	76	94	2	80	94	3		
W-19	W250 PA pw	1	PA	S	0.40	W	250	D	50	88	4	2	*		3	*	
W-20	W150 PC twill	1	PC	S	0.40	W	150	C	58	51	26	2	84	11	3	90	27
**KNITTED FABRICS**																	
K1-1	K1 125 P jersey	1	P	FT	0.32	K1	125		64	72	91	2	88	89	3	84	93
K1-2	K1 130 P eyelet	1	P	FT	0.50	K1	130		81	69	97	2	91	95	3	90	84
K1-3	K1 135 P jersey	1	P	FT	0.40	K1	135		57	56	89	2	82	83	3	80	85
K1-4	K1 275 P dbl knit	1	P	FT	1.00	K1	275		80	83	97	2	82	73	3	89	58
K1-5	K1 125 C jersey	1	C	S	0.35	K1	125		68	54	83	2	76	36	3	76	26
K1-6	K1 150 C jersey	1	C	S	0.50	K1	150		76	72	94	2	*		3	*	
K1-7	K1 190 C interlock	1	C	S	0.70	K1	190		82	73	98	2	61	93	3	79	79
K1-8	K1 175 PC interlock	1	PC	S	0.60	K1	175		70	73	87	2	84	89	3	69	74
K2-1	K2 50 P design A	1	P	F	0.25	K2	50		86	66	92	2			3		
K2-2	K2 100 P design H	1	P	F	0.25	K2	100		76	66	95	2	78	95	3		
K2-3	K2 125 P design D	1	P	F	0.25	K2	125		64	75	91	2			3		
K2-4	K2 200 P design F	1	P	F	0.60	K2	200		64	80	85	2			3		

* Results could not be obtained due to shedding or excessively low AP. K1 (weft-knits) are indicated as wpc and K2 (warp-knits) as cpc.

**Table 3:** Fabric structural detail and results of nonwoven fabrics tested in single layer.

Sample Ref.	Sample Description	Fabric Structure Detail					Results	
		Fibre Content	Thicknes (mm)	Structure	Weight (g/m^2^)	Porosity (%)	FE (%)	AP (%)
Nw-1	SbFw(n+bb)100 P/PP	P/PP	4.00	sb-fw(n)	100	98	75	92
Nw-2	Fw(bb)25 P	P	0.15	fw(bb)	25	88	71	94
Nw-3	Fw(bb)35 P	P	0.25	fw(bb)	50	86	62	98
Nw-4	Fw(bb)35 P	P	0.25	fw(bb)	50	86	83	89
Nw-5	Fw(bb)50 P	P	0.25	fw(bb)	50	86	64	81
Nw-6	Fw(bb)50 P	P	0.50	fw(bb)	50	93	69	91
Nw-7	Fw(bb)75 P	P	0.50	fw(bb)	75	89	85	97
Nw-8	Fw(n)100 P	P	2.00	fw(n)	100	96	85	86
Nw-9	Fw(n)100 P	P	2.00	fw(n)	100	96	86	95
Nw-10	Fw(n)100 P/PP	P/PP	1.00	fw(n)	100	85	75	80
Nw-11	Fw(n)125 P/PP	P/PP	2.00	fw(n)	125	95	83	99
Nw-12	Fw(n)75 P	P	0.25	fw(n)	75	78	69	91
Nw-13	Fw(ps)100 P	P	O.4	fw(ps)	100	82	81	82
Nw-14	Fw(ps)25 P	P	0.15	fw(ps)	25	88	62	96
Nw-15	Fw(ps)35 P	P	0.25	fw(ps)	50	86	77	99
Nw-16	Fw(ps)50 P	P	0.25	fw(ps)	50	86	75	88
Nw-17	Fw(ps)50 P	P	0.25	fw(ps)	50	86	85	92
Nw-18	Fw(ps)50 P	P	0.25	fw(ps)	50	86	86	95
Nw-19	Fw(ps)50 P	P	0.25	fw(ps)	50	86	86	89
Nw-20	Fw(ps)75 P	P	O.25	fw(ps)	75	78	80	76
Nw-21	Sb(ps)125 PP	PP	0.25	sb(ps)	125	74	88	88
Nw-22	Sb(ps)50 PP	PP	0.25	sb(ps)	50	90	62	95
Nw-23	Sb(ps)50 PP	PP	0.25	sb(ps)	50	90	88	85

## Data Availability

Not applicable.

## References

[1] Prather B.K.A., Wang C.C., Schooley R.T. (2020). Reducing transmission of SARS-CoV-2. Science.

[2] Milton D.K., Fabian M.P., Cowling B.J., Grantham M.L., McDevitt J.J. (2013). Influenza Virus Aerosols in Human Exhaled Breath: Particle Size, Culturability, and Effect of Surgical Masks. PLoS Pathog..

[3] Asadi S., Cappa C.D., Barreda S., Wexler A.S., Bouvier N.M., Ristenpart W.D. (2020). Efficacy of masks and face coverings in controlling outward aerosol particle emission from expiratory activities. Sci. Rep..

[4] WHO Advice on the Use of Masks in the Context of COVID-19: Interim Guidance-5 June 2020. https://apps.who.int/iris/handle/10665/3322932.

[5] Lindsley W.G., Beezhold D.H., Coyle J., Derk R.C., Blachere F.M., Boots T., Reynolds J.S., Mckinney W.G., Sinsel E., Noti J.D. (2021). Efficacy of universal masking for source control and personal protection from simulated cough and exhaled aerosols in a room. J. Occup. Environ. Hyg. ISSN.

[6] Davies A., Thompson K.A., Giri K., Kafatos G., Walker J., Bennett A. (2013). Testing the efficacy of homemade masks: Would they protect in an influenza pandemic?. Disaster Med. Public Health Prep..

[7] Anfinrud P., Bax C.E., Stadnytskyi V., Bax A. (2020). Could SARS-CoV-2 be transmitted via speech droplets?. medRxiv.

[8] Konda A., Prakash A., Moss G.A., Schmoldt M., Grant G.D., Guha S. (2020). Aerosol Filtration Efficiency of Common Fabrics Used in Respiratory Cloth Masks. ACS Nano.

[9] Howard J., Huang A., Li Z., Tufekci Z., Zdimal V., van der Westhuizen H.-M., von Delft A., Price A., Fridman L., Tang L.-H. (2020). Face Mask Against COVID-19: An Evidence Review. Br. Med. J..

[10] Duguid J.P. (1946). Expulsion of Pathogenic Organisms from Respiratory Tract. Br. Med. J..

[11] Chao C.Y.H., Wan M.P., Morawska L., Johnson G.R., Ristovski Z.D., Hargreaves M., Mengersen K., Corbett S., Li Y., Xie X. (2009). Characterization of expiration air jets and droplet size distributions immediately at the mouth opening. J. Aerosol Sci..

[12] Shah M., Crompton P., Vickers M.D.A. (1983). The efficacy of face masks. Ann. R. Coll. Surg. Engl..

[13] Leung N.H.L., Chu D.K.W., Shiu E.Y.C., Chan K.H., McDevitt J.J., Hau B.J.P., Yen H.L., Li Y., Ip D.K.M., Peiris J.S.M. (2020). Respiratory virus shedding in exhaled breath and efficacy of face masks. Nat. Med..

[14] Xie X., Li Y., Sun H., Liu L. (2009). Exhaled droplets due to talking and coughing. J. R. Soc. Interface.

[15] Dbouk T., Drikakis D. (2020). On respiratory droplets and face masks. Phys. Fluids.

[16] Scharfman B.E., Techet A.H., Bush J.W.M., Bourouiba L. (2016). Visualization of sneeze ejecta: Steps of fluid fragmentation leading to respiratory droplets. Exp. Fluids.

[17] Asadi S., Gaaloul ben Hnia N., Barre R.S., Wexler A.S., Ristenpart W.D., Bouvier N.M. (2020). Influenza A virus is transmissible via aerosolized fomites. Nat. Commun..

[18] Zhong W. (2013). An Introduction to Healthcare and Medical Textiles.

[19] Department of Trade Industry and Competition Recommended Guidelines for the Manufacturing of Fabric Face Masks for General Public Use. http://www.treasury.gov.za/comm_media/press/2020/Annexure%20B%20-%20Recommended%20Guidelines%20Fabric%20Face%20Masks%20RSA%20DTIC.pdf.

[20] Hinds W.C. (1999). Aerosol Technology: Properties, Behavior and Measurement of Airborne Particles.

[21] Podgórski A., Bałazy A., Gradoń L. (2006). Application of nanofibers to improve the filtration efficiency of the most penetrating aerosol particles in fibrous filters. Chem. Eng. Sci..

[22] Abdul-Rahman A., Pilouk M., Aebischer B., Catenazzi G., Jakob M., Africa W., Appiah S.K., Ageep T.B., Cox J., Hassan M.M. (2012). Thermal Comfort. Geophys. Res. Lett..

[23] Tcharkhtchi A., Abbasnezhad N., Zarbini Seydani M., Zirak N., Farzaneh S., Shirinbayan M. (2021). An overview of filtration efficiency through the masks: Mechanisms of the aerosols penetration. Bioact. Mater..

[24] Gupta D. (2011). Functional clothing- definition and classification. Indian J. Fibre Text. Res..

[25] Kadolph S., Marcketti S. (2016). Textiles.

[26] Militky J., Novak O., Kremenakova D., Wiener J., Venkataraman M., Zhu G., Yao J., Aneja A. (2021). A review of impact of textile research on protective face masks. Materials.

[27] Anand S.C., Lawton P.J. (1991). The development of knitted structures for filtration. J. Text. Inst..

[28] Horrocks A.R., Anand S.C. Handbook of Technical Textiles.

[29] Çinçik E., Koç E. (2012). An analysis on air permeability of polyester/viscose blended needle-punched nonwovens. Text. Res. J..

[30] Bunsell A.R. (2018). Handbook of Properties of Textile and Technical Fibres.

[31] WHO Coronavirus Disease (COVID-19) Advice for the Public: When and How to Use Masks. https://www.who.int/emergencies/diseases/novel-coronavirus-2019/advice-for-public/when-and-how-to-use-masks.

